# The diagnostic and prognostic role of RhoA in hepatocellular carcinoma

**DOI:** 10.18632/aging.102110

**Published:** 2019-07-22

**Authors:** Yi Bai, Fucun Xie, Fei Miao, Junyu Long, Shan Huang, Hanchun Huang, Jianzhen Lin, Dongxu Wang, Xu Yang, Jin Bian, Jinzhu Mao, Xi Wang, Yilei Mao, Xinting Sang, Haitao Zhao

**Affiliations:** 1 Department of Liver Surgery, Peking Union Medical College Hospital, Chinese Academy of Medical Sciences and Peking Union Medical College (CAMS & PUMC), Beijing, China; 2 Department of Statistics, Tianjin University of Finance and Economics Pearl River College, Tianjin, China; 3 Department of Immunology, Beijing Key Laboratory for Cancer Invasion and Metastasis, Advanced Innovation Center for Human Brain Protection, School of Basic Medical Sciences, Capital Medical University, Beijing, China

**Keywords:** RhoA, hepatocellular carcinoma, expression level, prognostic biomarker, diagnostic biomarker, aging, age-related diseases

## Abstract

The aim of this study was to investigate the expression level of Ras homolog gene family, member A (RhoA) in patients with hepatocellular carcinoma (HCC) and to investigate the prognostic and diagnostic value of RhoA. Data mining from various data bases and wet experiments on samples from Peking Union Medical College Hospital showed that *RhoA* mRNA and protein expression were significantly higher in the HCC tissues than in the normal tissues. Higher expression at both the mRNA and protein levels was associated with a poorer prognosis. High sensitivity (92.5%) and specificity (90.0%) were observed in the diagnostic model based on protein level rather than mRNA level. *RhoA* expression was modulated by genetic amplification. The lysosome, pathogenic *Escherichia coli* infection, purine metabolism and pyrimidine metabolism pathways were mainly enriched in the high *RhoA* level group, while the hedgehog signaling, linoleic acid metabolism, olfactory transduction and taste transduction pathways were enriched in the low RhoA level group. RhoA is commonly upregulated in HCC tissues, and its expression at both the mRNA and protein levels is associated with poor prognosis. Notably, RhoA protein levels serve as a diagnostic biomarker for HCC.

## INTRODUCTION

In different countries, the incidence rates (cases/100,000 people) of hepatocellular carcinoma (HCC) range from 1.9 to 41.3 among males and from 0.8 to 13.9 among females [1]. Globally, HCC is the second most common cause of cancer-related death and is estimated to have been responsible for nearly 745,000 deaths in 2012 [2]. Regarding the diagnosis and screening of HCC, although there are multiple traditional methods, including ultrasound imaging (UI), computer tomography (CT) imaging, magnetic resonance imaging (MRI) and the measurement of serum alpha-fetoprotein (AFP) levels and other potential biomarkers, such as glypican-3 (GPC3), dickkopf-1 (DKK1) and circulating miRNAs, early diagnosis with high specificity and sensitivity remains difficult; 70–80% of patients are in advanced stages by the time they present symptoms and therefore miss the opportunity to receive radical resection [3], which is especially common in China [4]. Consequently, the five-year overall survival (OS) rate of HCC patients after initial diagnosis is lower than 20%. Therefore, developing a better biomarker with high specificity and sensitivity for early diagnosis and prognosis is important.

Ras homolog gene family, member A (RhoA) is a small GTPase protein in the Rho family containing two switch regions, Switch I and Switch II, whose conformational states are modified following the activation or deactivation of the protein. The key amino acids of RhoA at Gly14, Thr19, Phe30, and Gln63, which are involved in the stabilization and regulation of guanosine triphosphate (GTP) hydrolysis, are highly conserved. RhoA is primarily involved in actin organization, myosin contractility, cell cycle maintenance, cellular morphological polarization, cellular development, and transcriptional control. Accumulated evidence has demonstrated that RhoA was closely associated with cancer in relation to venous invasion, microscopic satellite lesions, advanced pTNM stage, progression [5], cell differentiation [6] and disease-free survival rates [7]. However, the prognostic and diagnostic value of RhoA in HCC is still unclear, and further research is urgently needed.

Here, using bioinformatics data mining, we found that both the gene and protein levels of RhoA were differentially expressed between liver cancer tissues and adjacent normal tissues, which indicated the potential of *RhoA* as a diagnostic biomarker. Analysis of The Cancer Genome Atlas (TCGA) data suggested that higher *RhoA* gene expression was associated with poorer prognosis, and similar patterns were also observed in two additional Gene Expression Omnibus (GEO) cohorts. The protein expression, but not the mRNA level, of RhoA varied greatly between liver cancer tissues and normal tissues, making it possible to build not only a prognostic model but also a diagnostic model, which was validated in a Peking Union Medical College Hospital (PUMCH) cohort with 30 normal tissue samples and 134 HCC samples.

## RESULTS

### mRNA and protein expression profile of RhoA in the HPA

**Figure 1:**
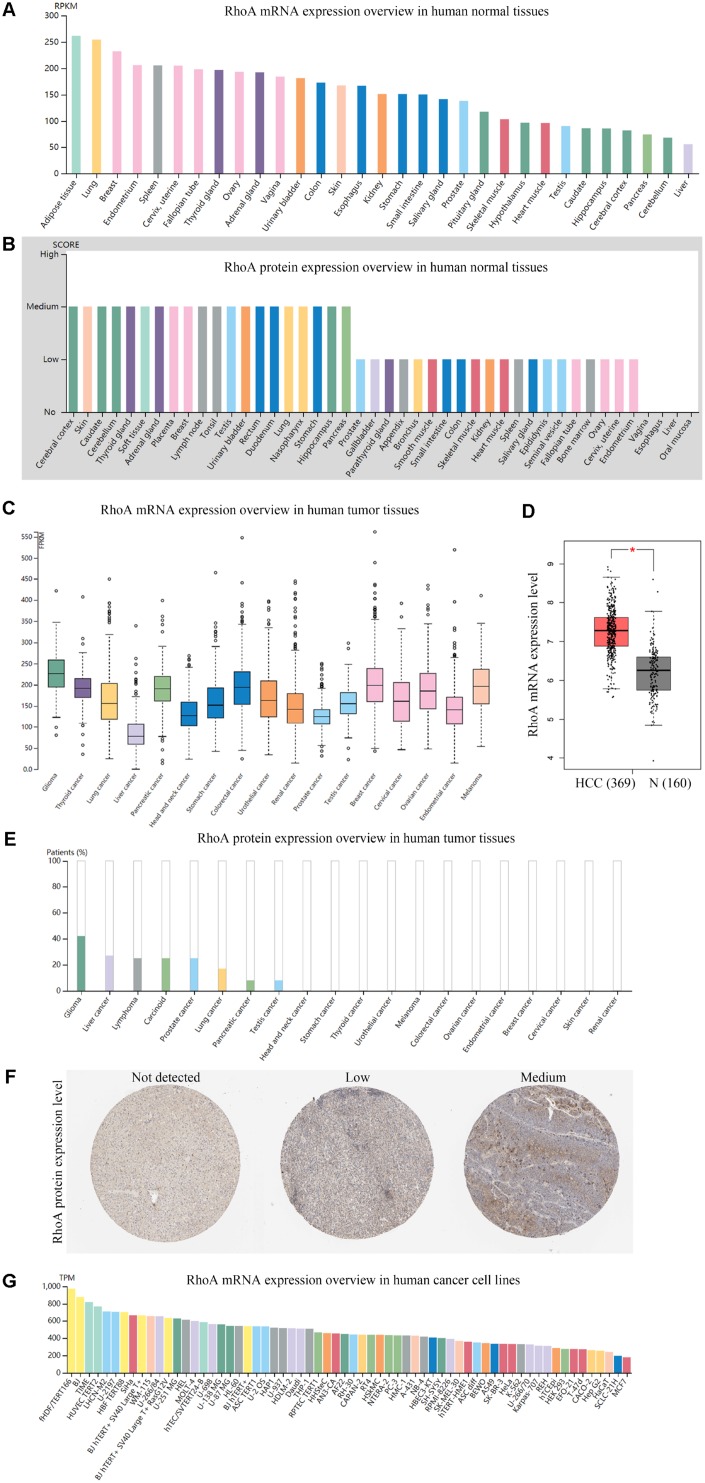
**Gene and protein expression profiles of RhoA in tissue samples and cancer cell lines.** *RhoA* mRNA expression (**A**) data from the GTEx dataset in the Human Protein Atlas) and protein expression (**B**) data from the Human Protein Atlas) in normal human tissues. *RhoA* gene expression in common human tumor tissues (**C**) data from the TCGA dataset in the Human Protein Atlas). Comparison of *RhoA* mRNA expression between hepatocellular carcinoma tissues and normal liver tissues, including normal TCGA and GTEx data (**D**) data from GEPIA). RhoA protein expression overview in human tumor tissues (**E**) data from the TCGA CAB005052 dataset in the Human Protein Atlas) and representative immunohistochemistry (IHC) images (**F**) pictures from the Human Protein Atlas) with RhoA antibody (1:25, Cat#1600-1, Abcam, Cambridge, UK). *RhoA* mRNA expression in human cancer cell lines (**G**, data from RNA cell line category in the Human Protein Atlas).

By examining the *RhoA* expression profile in the HPA, we found that the RNA expression of *RhoA* in the normal liver tissue samples was the lowest compared with that in the other 30 human tissue samples (Figure 1A), and the protein level of RhoA was nearly undetectable in hepatic tissues (Figure 1B). As Figure 1C shows, the level of *RhoA* in the HCC tissue samples was relatively low among the 17 carcinoma types; however, the expression of RhoA was found to be significantly upregulated in HCC tissues compared with that in normal tissues (Figure 1D). Interestingly, regarding RhoA protein expression, liver cancer exhibited a high positive rate, which ranked second among the positive expression rates of 20 common cancer types (Figure 1E). Figure 1F displays representative IHC images of the distinct RhoA expression levels, including undetected, low, and medium. In addition, Hep G2, which is a frequently used hepatic carcinoma cell line, had a similar *RhoA* expression pattern as that of the liver cancer tissue shown in Figure 1C, as identified by the Cancer Cell Line Encyclopedia (CCLE) web searching tool. Hence, the RhoA protein level, rather than the gene expression level, may be a more sensitive biomarker to diagnose HCC.

### Prognostic role of *RhoA* gene expression in liver cancer patients

**Figure 2:**
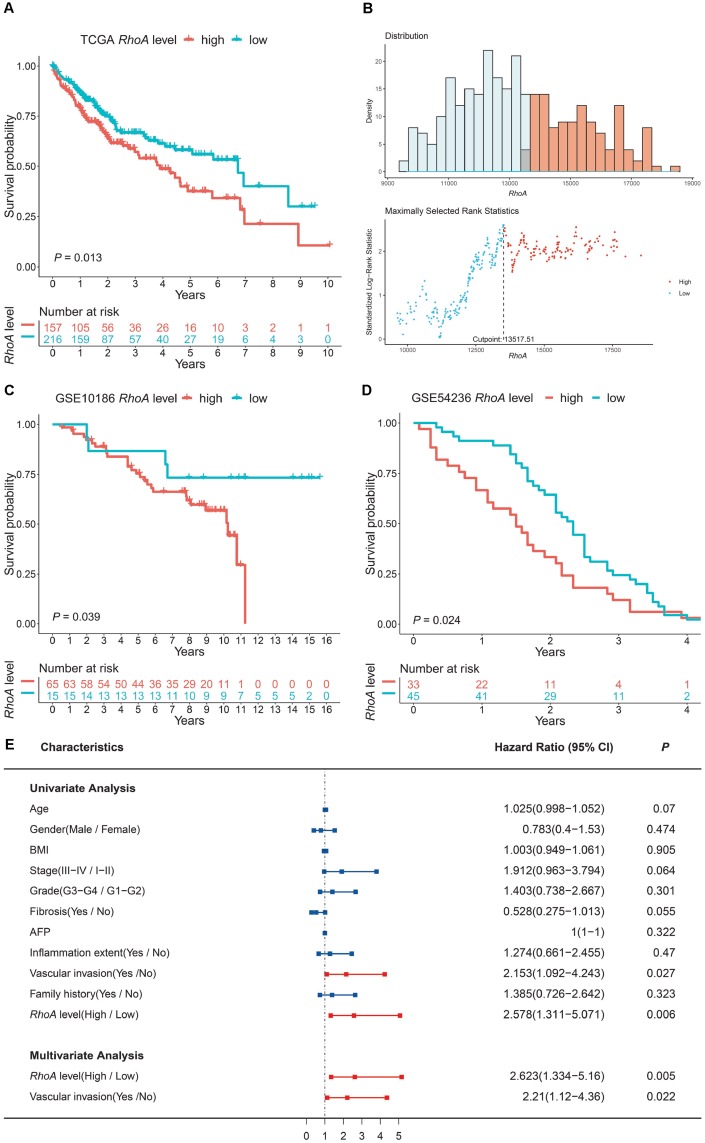
***RhoA* expression level as an independent prognostic factor in HCC.** The high expression level of *RhoA* suggests poor prognosis based on the TCGA training set (**A**), and the optimal cutoff point was calculated via the X-tile method (**B**). High *RhoA* expression levels were also unfavorable in two GEO validation sets (**C**, GSE10186; **D**, GSE54236). Univariate and multivariate Cox regression analyses of clinical indicators and *RhoA* levels related to prognosis: red bars represent prognostic factors, and blue bars represent nonprognostic factors (**E**).

To explore the association between *RhoA* mRNA expression and OS in liver cancer patients, we performed Kaplan-Meier survival analysis based on data from TCGA. The results indicated that the HCC patients with high *RhoA* expression had poorer prognoses (Figure 2A). Notably, the optimal cutoff point of *RhoA* mRNA expression was calculated based on the X-tile method, which can produce the optimal cutoff value to predict survival time (Figure 2B). Moreover, the tumor-promoting role of *RhoA* was also demonstrated by two genomic spatial event (GSE) datasets (GSE10186 and GSE 54236), in which the *P* values were 0.039 and 0.024, respectively (Figure 2C and Figure 2D).

To further investigate the independent prognostic value of *RhoA*, univariate and multivariate Cox regression analyses were conducted. As shown in Figure 2E, vascular invasion (HR: 1.274; 95% CI: 1.092–4.243, *P* = 0.027; Figure 2E) and high *RhoA* levels (HR: 2.578; 95% CI: 1.311–5.071, *P* = 0.006; Figure 2E) were associated with poor OS in the univariate analysis. In addition, multivariate analysis also demonstrated that the aforementioned factors were independent risk factors of OS (*P* < 0.05; Figure 2E).

### *RhoA* expression is associated with gender

**Figure 3:**
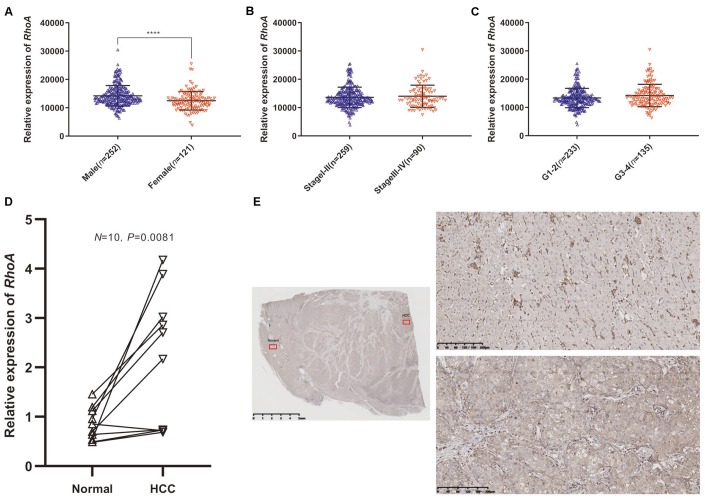
***RhoA* mRNA expression in different groups of patients and differential expression verification.** Relative expression of *RhoA* in males and females (**A**), stage I-II and stage III-IV (**B**), and grade 1–2 and grade 3–4 (**C**) from TCGA data. The relative *RhoA* expression in ten pairs of normal tissues and HCC tissues from Peking Union Medical College Hospital identified through real-time quantitative polymerase chain reaction (**D**). The representative immunohistochemistry images of HCC tissue (lower right) and adjacent normal tissue (upper right): The red squares shown in the figure represents the enlarged area. (**E**).

Next, we divided HCC patients from TCGA into a high-risk group and a low-risk group according to the optimal cutoff value of *RhoA* expression mentioned before to explore the relationships of *RhoA* expression with different clinicopathological parameters, and we only found that male patients had significantly higher *RhoA* gene expression levels than female patients (Table 1, Supplementary Table 1, and Figure 3A). However, the other clinical characteristics, such as different stage levels or grade levels of HCC, seemed to have no influence on *RhoA* expression levels (Table 1, Supplementary Table 1, Figure 3B, 3C).

**Table 1:** The association between RhoA expression and the clinical parameters of patients with liver cancer.

	***RhoA* level**			
**Categorical variable**	**Low(N=216)**	**High(N=157)**	**Chi-square or *F* value**	***P* value**
**Gender**				
Female	86	35	11.949	0.0005467
Male	130	122		
**Stage**				
I-II	153	106	0.21049	0.6464
III-IVB	50	40		
**Grade**				
G1-G2	143	90	2.3593	0.1245
G3-G4	71	64		
**Fibrosis**				
No	48	27	1.8842	0.1699
Yes	74	65		
**Inflammation extent**				
No	74	44	1.1202	0.2899
Yes	65	53		
**Vascular invasion**				
No	119	89	0.20509	0.6506
Yes	66	43		
**Family history**				
No	125	84	0.11809	0.7311
Yes	64	48		
**Continuous variables**				
Age	60(58, 62)	59(57, 61)	0.75	0.387
BMI	25(24, 26)	26(25, 27)	1.69	0.195
AFP	4980(1283, 8677)	20152(−4890,45195)	0.992	0.32

BMI: body mass index; AFP: alpha fetoprotein.

### mRNA and protein expression validation of *RhoA*

We further extracted RNA from 10 frozen HCC and paired adjacent liver tissues to quantify *RhoA* gene expression. The HCC tissues did have a significantly higher *RhoA* expression, which was consistent with the pattern identified in the HPA. (Figure 3D). Moreover, the IHC test of RhoA showed that the hepatic cells in the normal tissue were mainly stained negatively except for portions of the interstitial substance (Figure 3E, upper parts), while tumor cells in the HCC tissue from the same section were stained positively (Figure 3E, lower parts). In summary, although the mRNA and protein levels of RhoA were significantly higher in the HCC tissues than in the normal adjacent tissues, the RhoA protein level, rather than the mRNA level, seems to have a better distinction ability, which indicates its potential as a diagnostic biomarker.

### Diagnostic model based on RhoA protein levels in liver cancer patients

**Figure 4:**
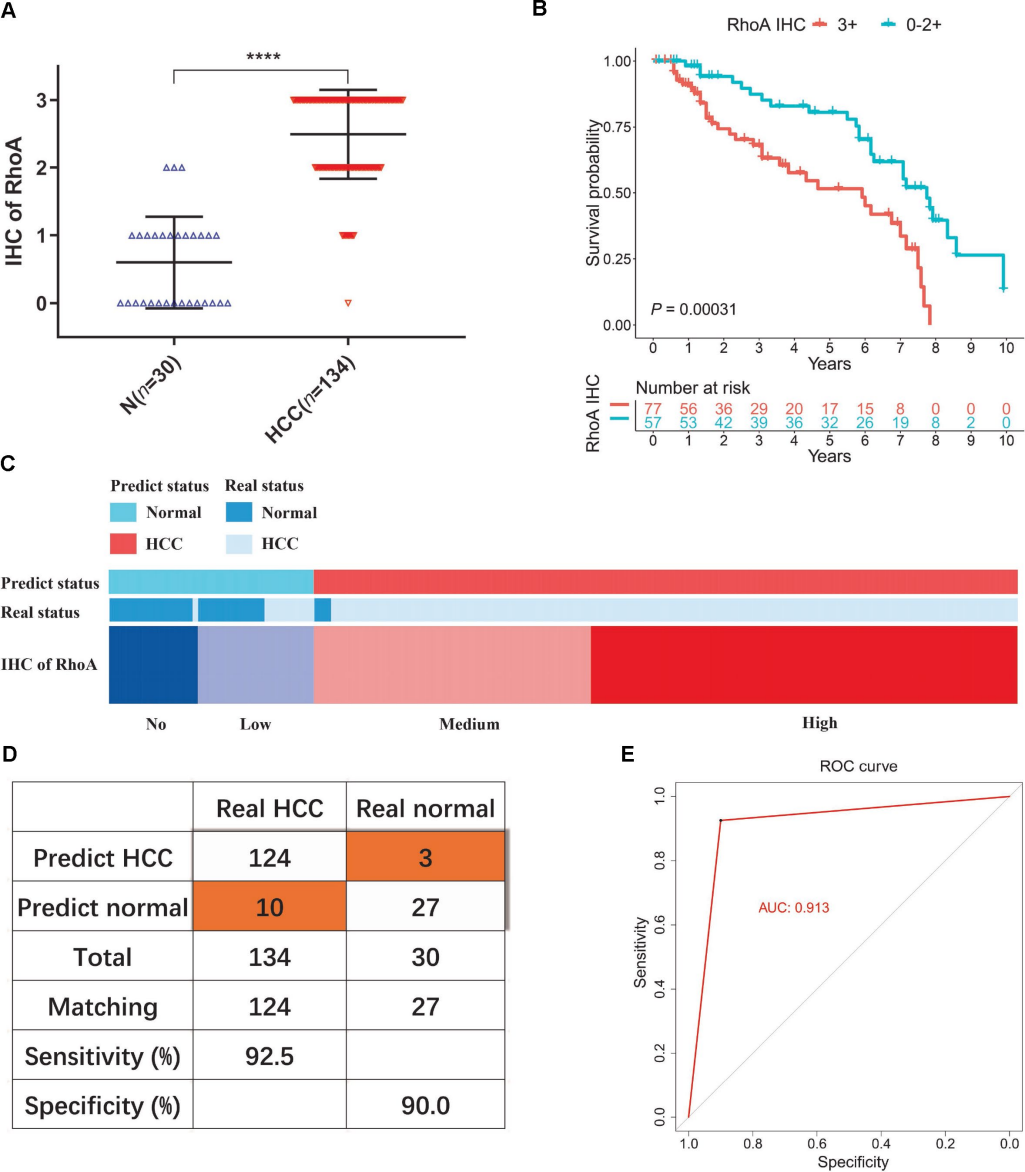
**Diagnostic model of RhoA protein expression in liver cancer patients.** The staining intensities of RhoA via immunohistochemistry chips from PUMCH patient samples (**A**). Kaplan-Meier curves of overall survival (**B**) of liver cancer patients with high RhoA protein expression levels (3+) and low RhoA protein expression levels (0–2+). Diagram (**C**), sensitivity and specificity validation (**D**) and receiver operating characteristic curve (**E**) of the diagnostic model according to RhoA immunohistochemistry level.

To test our hypothesis, we performed additional IHC staining with 30 normal tissue samples and 134 HCC tissue samples from an HCC patient cohort from PUMCH to detect the protein expression profile of RhoA. Consistent with the previous results, the HCC samples had significantly higher RhoA protein levels (Figure 4A). Next, we divided patients into two groups, one with a high RhoA protein level (3+) and the other with a low level (0−2+). Similar to the outcome of the analysis of the prognostic function of *RhoA* gene expression, the HCC patients with longer OS times had lower RhoA protein levels (Figure 4B). Considering that the protein level of RhoA has better tissue specificity than gene expression between liver tumor tissues and adjacent normal tissues, the RhoA protein expression based on the results of the IHC analysis of normal tissues was mainly undetected (0) or low (1+), while that of the cancerous tissues was usually medium (2+) or high (3+). We proposed a diagnostic model based on this phenomenon (Figure 4C). After validation, the sensitivity and specificity of the model were 92.5% and 90.0%, respectively (Figure 4D). The area under the curve (AUC) of the receiver operating characteristic (ROC) curve was 0.913 (Figure 4E), which indicates the good performance of this model. To exclude false positive caused by antibody non-specificity, we performed immunohistochemical tests on some samples with another RhoA antibody (1:50, AF6352, Affinity biosciences, USA) and the results are in line with previous one (data not shown). Overall, the diagnostic model based on the RhoA IHC results expanded our current detection approaches for HCC.

### *RhoA* expression is modulated by genetic amplification and related KEGG pathways

**Figure 5:**
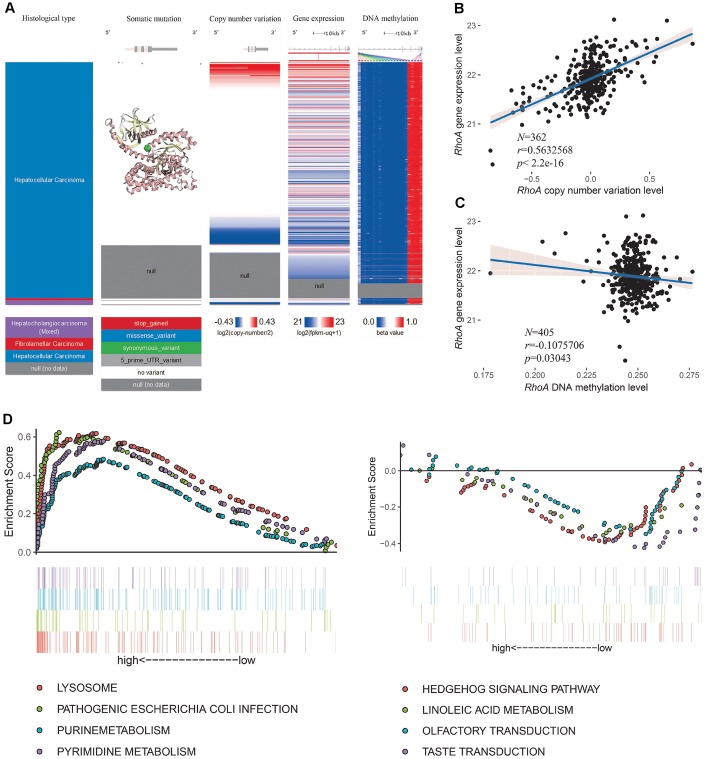
***RhoA* dysregulation and related KEGG analysis.** Multiomic data of *RhoA* in liver cancer tissues are displayed in a heatmap (**A**). The correlation between *RhoA* gene expression level and copy number variation level (**B**) or DNA methylation level (**C**) were determined by regression analysis. The top 4 Kyoto Encyclopedia of Genes and Genomes pasthways identified via gene set enrichment analysis of tissues with high and low *Rho* expression levels (**D**).

Next, we explored the mechanisms of *RhoA* dysregulation using deep sequencing data from the University of California, Santa Cruz. After visualizing the somatic mutation, copy number variation, DNA methylation, and expression of *RhoA*, we found only four HCC samples with single nucleotide polymorphisms (SNPs), which demonstrated that point mutation did not play an essential role in *RhoA* expression (Figure 5A). Notably, the copy number variation and gene expression of *RhoA* shared a coexpression pattern, and the significant association was further validated via regression analysis (*r* = 0.563, *P* < 0.05, Figure 5B). However, the DNA methylation profile of the HCC tissues showed little variation (Figure 5A). To depict the exact relationships between DNA methylation and gene expression levels, we analyzed 405 samples with both methylation data and gene expression levels. As shown in Figure 5C, the increase in *RhoA* DNA methylation only slightly attenuated *RhoA* gene expression (*r* = −0.108, *P* < 0.05, Figure 5C). Furthermore, to detect the primary KEGG pathways in the two groups (*RhoA* high and low), we performed GSEA and found that samples with high levels of RhoA were enriched with genes mainly involved in the lysosome, pathogenic *Escherichia coli* infection, purine metabolism and pyrimidine metabolism pathways (Figure 5D, left part), while those with low levels of *RhoA* had predominant genes involved in the hedgehog signaling pathway, linoleic acid metabolism, olfactory transduction and taste transduction (Figure 5D, right part).

Therefore, it seems that the copy number amplification level of *RhoA*, rather than the decrease in the DNA methylation level, plays a major role in the overexpression of *RhoA* in HCC tissues.

## DISCUSSION

As one of the leading causes of cancer deaths around the world, liver cancer continues to exhibit increasing incidence and mortality rates, especially in East Asia, Southeast Asia, Africa and southern Europe [8, 9]. Radical surgery can significantly improve the prognosis of some patients with early stage HCC; however, due to a lack of effective screening approaches and noticeable symptoms, most patients are in advanced stages the time of diagnosis and miss the optimal opportunity for treatment, which leads to dismal prognosis. Consequently, considerable research efforts have been devoted to identifying diagnostic and prognostic biomarkers of HCC with high specificity and sensitivity.

RhoA is one of the prototypical members of mammalian Rho GTPases, which include 23 intracellular signaling molecules, such as Ras-related C3 botulinum toxin substrate 1 (RAC1) and cell division cycle 42 (CDC42) [10–12]. The change in Rho GTPase proteins between the GTP-bound active form and the GDP-bound inactive form is regulated by three sets of molecules: guanine nucleotide exchange factors (GEFs), GTPase-activating proteins (GAPs), and guanine nucleotide dissociation inhibitors (GDIs) [10–12]. Activated Rho GTPases interact with downstream proteins and regulate cytoskeletal dynamics and a variety of biological processes, including cell division, survival, migration, and adhesion [10–12]. Considering the crucial roles of RhoA in the regulation of cell morphology, motility, and cell-cell and cell-matrix adhesion, we can easily deduce that RhoA is associated with carcinoma metastasis, which is one of the leading causes of death in patients with solid tumors [13, 14]. Recent studies have reported that the invasiveness and metastasis of rat and human hepatoma cells [15, 16], bladder cancer cells [17], colorectal cancer cells [18], and lung cancer cells [19] can be suppressed by inhibiting the Rho-ROCK (Rho-kinase) signaling pathway. However, there is some contradictory evidence showing that RhoA inactivation promoted the migration and metastasis of triple-negative breast cancer [20] and increased tumor growth and metastasis in colorectal cancer [21], liver cancers [22], and nasopharyngeal carcinoma [13]. Further mechanistic studies are needed to better elucidate the regulation of the RhoA signaling pathway in tumor metastasis, especially in HCC.

Taking the functions of RhoA in essential signaling pathways into account, we speculate that RhoA may be a potential functional biomarker in HCC. We initially examined both the mRNA and protein expression patterns of RhoA in normal human organs, common cancer types and cell lines using an HPA web-based tool. Intriguingly, although *RhoA* was significantly upregulated in HCC tissues compared with its expression in normal liver tissues, the basal expression of *RhoA* in normal liver tissue, HCC tissue, and the HCC cell line Hep G2 was actually very low among all the specimens. Notably, the RhoA protein was not detected in normal liver tissues but was significantly enriched in most HCC samples. It is well accepted that protein is a function executor according to the central dogma. The RhoA protein expression level in HCC ranked second, next to that in glioma, in all common tumor types, which indicated that the RhoA protein, rather than the gene, may promote liver tumorigenesis as an essential functional element. Recent studies have suggested that RhoA is one of the genes that is most frequently overexpressed in various cancer cells and is involved in cell division processes, and *RhoA* promotes HCC growth through the RhoA/F-actin/Hippo-YAP signaling axis [23]. In addition, the coordinated expression of Rac GTPase-activating protein 1 (RACGAP1) and epithelial cell transforming sequence 2 (ECT2) upregulated RhoA activity in HCC cells [23–25].

To evaluate the prognostic role of *RhoA*, we subsequently performed Kaplan-Meier analysis in the TCGA HCC cohort and found that high *RhoA* expression correlated well with poor prognosis. Two additional GEO cohorts also demonstrated this phenomenon. In addition, after univariate and multivariate analyses, *RhoA* expression levels, together with vascular invasion, were both independent prognostic factors in HCC, which was consistent with the results of a previous study [6]. Recent studies have also proven the prognostic value of RhoA in other cancer types, such as colorectal cancer, breast cancer, and glioma. Colorectal cancer patients with positive RhoA protein expression had higher vascular invasion rates, higher clinical stages, and lower 5-year survival rates [26]. Breast cancer samples with phosphorylated RhoA (P-RhoA) were associated with poorer prognosis [27]. Lin Yu demonstrated that SND1 (Staphylococcal nuclease domain-containing protein 1) and RhoA were independent predictors of poor prognosis in glioma patients [28]. Our results suggested only a relationship between gender and *RhoA* gene expression.

Furthermore, RhoA protein expression may be a diagnostic biomarker in HCC because its high liver cancer specificity was demonstrated by analyzing IHC sections from tumor resection margin areas of 9 HCC patients from PUMCH. Hence, we estimated the RhoA IHC staining intensities in a PUMCH HCC cohort of 130 liver tumor samples and 30 adjacent normal samples. The role of the RhoA protein was apparent from its expression and distribution. In addition to the prognostic ability of the gene, the RhoA protein level can clearly distinguish between normal and cancerous tissues with high sensitivity and specificity. This is the first time that the diagnostic role of RhoA was proposed and proven.

The essential position occupied by RhoA in the cancer cellular signaling pathway network has emphasized the importance of RhoA in cancer pathophysiology, and the intrinsic characteristics of RhoA make RhoA a great potential distinguished diagnostic and prognostic biomarker for liver cancer. As described before, instead of acting solely as a cancer indicator, RhoA also acts as a functional regulator. We next explored the dysregulation mechanism of gene expression via multiomics analysis. The results showed that the copy number amplification of *RhoA* mainly contributed to its increased gene expression, which may be a potential therapeutic target for HCC. The predominant KEGG pathways in the high *RhoA* level group and the low *RhoA* level group were further identified by using GSEA to reveal potential differential signaling pathways.

Overall, our study confirmed RhoA mRNA and protein levels as prognostic biomarkers in HCC through the analysis of multiple cohorts, and the use of RhoA as a prognostic biomarker has been reported in previous studies [5, 6, 23]. To the best of our knowledge, this is the first time that a diagnostic model was proposed based on RhoA IHC staining results rather than gene expression. Our estimations indicate that this model has very good performance (AUC=0.913) and high sensitivity and specificity (92.5% and 90.0%, respectively). However, due to limited HCC cohorts and RhoA IHC expression results, we could not perform external verification of this diagnostic model. In addition, future work will focus on utilizing clinical parameters along with biomarkers, which may improve the performance of the biomarkers [29]. In addition, further clarification of the exact RhoA expression regulatory mechanism is urgently needed.

## MATERIALS AND METHODS

### Bioinformatics data mining and processing

The mRNA level and protein level of RhoA in both normal human tissues and cancerous tissues, as well as gene expression in cancer cell lines, were examined using data from the Human Protein Atlas (HPA) (http://www.proteinatlas.org/) [30, 31]. Representative immunohistochemistry images of RhoA protein expression profiles in HCC samples with different staining intensities were also downloaded from the HPA. The differential mRNA expression level of RhoA between HCC tissues and normal tissues from the TCGA database and the Genotype-Tissue Expression (GTEx) project were analyzed via the Gene Expression Profiling Interactive Analysis (GEPIA) web tool (http://gepia.cancer-pku.cn/). The mRNA transcriptome data and clinical parameters of liver hepatocellular carcinoma (LIHC) patients were obtained from the TCGA data portal (https://portal.gdc.cancer.gov/). Two other HCC validation datasets (GSE10186 and GSE54236) containing mRNA expression data and corresponding survival times were retrieved and downloaded from the GEO database (https://www.ncbi.nlm.nih.gov/geo/). Histological type, somatic mutation, copy number variation, gene expression, and DNA methylation of *RhoA* in liver cancer were visualized via a heatmap generated from the Xena browser web tool (https://xenabrowser.net/), and data used to analyze the factors affecting *RhoA* gene expression were downloaded from the Xena browser web tool. Analyzing the data downloaded from the TCGA and GEO databases did not require the approval of an ethics committee. Gene Set Enrichment Analysis (GSEA) was conducted to compare the distinct Kyoto Encyclopedia of Genes and Genomes (KEGG) pathways between the high RhoA level group and the low RhoA level group based on the TCGA HCC mRNA expression data.

### Experimental validation with samples from Peking Union Medical College Hospital

From January 2004 to August 2016, thirty paired HCC and para-cancerous normal tissues and 104 HCC tissues from patients who underwent surgery and pathologically diagnosed as HCC at PUMCH were collected and made into four formalin-fixed paraffin-embedded (FFPE) tissue chips, the remaining tissues were frozen in liquid nitrogen and then stored at −80°C. Among these patient samples, the differential RhoA expression of ten frozen HCC tissues and paired adjacent normal tissues were validated by quantitative polymerase chain reaction (qPCR) as described previously [32]. The primer sequences were as follows: RhoA forward primer: 5′-AGCCTGTGGAAAGACATGCTT-3′; RhoA reverse primer: 5′-TCAAACACTGTGGGCACATAC-3′; β-actin forward primer: 5′-GCCGGGACCTGACTGACTAC-3′; β-actin reverse primer: 5′-CGGATGTCCACGTCACACTT-3′. Immunohistochemistry (IHC) sections of four FFPE tissue chips were stained with the RhoA antibody (EPR18134, RabMAb, UK) at a dilution of 1:100 to detect their protein profiles. In addition, nine IHC sections of the cancer margin with both normal and tumor components were used to elucidate the differential protein expression level of RhoA. The study was approved by the Medical Ethics Committee of PUMCH of the Chinese Academy of Medical Sciences (CAMS) & PUMC, and all patients signed informed consent forms.

### Immunohistochemistry

Paraffin slices are cut into 3μm thick and placed on anti-unloading slides, baking 1 hour at 72°C. After 2–3 dewaxing with dimethylbenzene, slides are immersed into gradient alcohol (100%, 100%, 95% and 75%) successively. Rinse with PBS (1‰ Tween 20) for 5 times. Not less than 2 minutes each time. Preheat the repair solution in the pressure cooker to boiling then put the slides into the repair solution. After 2.5 minutes, wash slides to room temperature with cool water. Rinse with PBS for 5 times. Incubate with 3% H_2_O_2_ 10–15 minutes at room temperature. Wash with distilled water first, then PBS for 5 times. Add primary antibody and overnight at 4 °C. Rinse with PBS for 5 times. Incubate with one-step detection system (PV-8000, ZSGB-BIO, China) for 20 minutes at 37°C. Rinse with PBS for 5 times. Color development with DAB (ZLI-9018, ZSGB-BIO, China) 6–8 minutes. Termination reaction with water. Re-satin with hematoxylin and wash with water. Differentiated by hydrochloric alcohol quickly. Return blue for 3–5 minutes via repair solution and rinse with running water for 3–5 minutes. Seal slides after dehydration with gradient alcohol.

Interpretation of immunohistochemical results was performed by two independent experienced pathologists based on the proportion of positive cells without considering dyeing intensity. Histochemical score from 0 to 3 as follows: 0, 1–5% positive cells; 1, 6–25% positive cells; 2, 26–75% positive cells; and 3, >76% positive cells. Samples with discordant scores from the two pathologists were discussed and re-scored.

### Statistical analysis

Statistical analysis was performed using R software v.3.5.2. Continuous variables are reported as the means ± standard deviations (SDs). Differences between groups were compared by unpaired Student’s *t*-test and visualized by GraphPad Prism v.8.0 (GraphPad Inc.). The associations between *RhoA* expression and clinicopathological parameters were evaluated using the *χ*^2^ test. The OS curves of the above three HCC patient cohorts were generated based on the best cutoff value (X-tile algorithm) with the survival package in R. Univariate and multivariate Cox regression analyses were performed to identify independent prognostic variables based on *RhoA* level and other clinical characteristics, including age, gender, body mass index (BMI), stage, grade, fibrosis, AFP, extent of inflammation, vascular invasion, and family history. Unless noted otherwise, *P* < 0.05 was considered statistically significant. Linear regression analyses were conducted to assess the correlations between *RhoA* gene expression and copy number variation and between *RhoA* gene expression and DNA methylation. |*R*|>3 and *P* < 0.05 were considered relevant and statistically significant.

## CONCLUSIONS

*RhoA* is commonly upregulated in HCC tissues, and both high mRNA expression and high protein expression levels are associated with poor prognosis. Notably, RhoA protein levels serve as a diagnostic biomarker for HCC.

## AUTHOR CONTRIBUTIONS

YB and FCX conceived the study and performed bioinformatics analyses. JZL, DXW, XY, JB, and FM downloaded and organized the clinical and gene expression data. YB and SH designed and executed experiments. FCX, FM, JYL, JZM and HCH performed the statistical analyses. YB and FCX wrote the manuscript. XW, YLM, XTS, and HTZ critically revised the article for essential intellectual content and provided administrative support. All authors read and approved the final version of the manuscript. All authors also reviewed and revised the manuscript. HTZ was the guarantor of this study.

## Supplementary Material

Supplementary Figure 1

Supplementary Table 1
