# Supplementary figures and images for: The diagnostic and prognostic role of RhoA in hepatocellular carcinoma
Source: Aging (Albany NY). 2019 Jul 22;11(14):5158–72. doi: 10.18632/aging.102110 (PMC6682515; doi:10.18632/aging.102110)

## SUPPLEMENTARY FIGURE

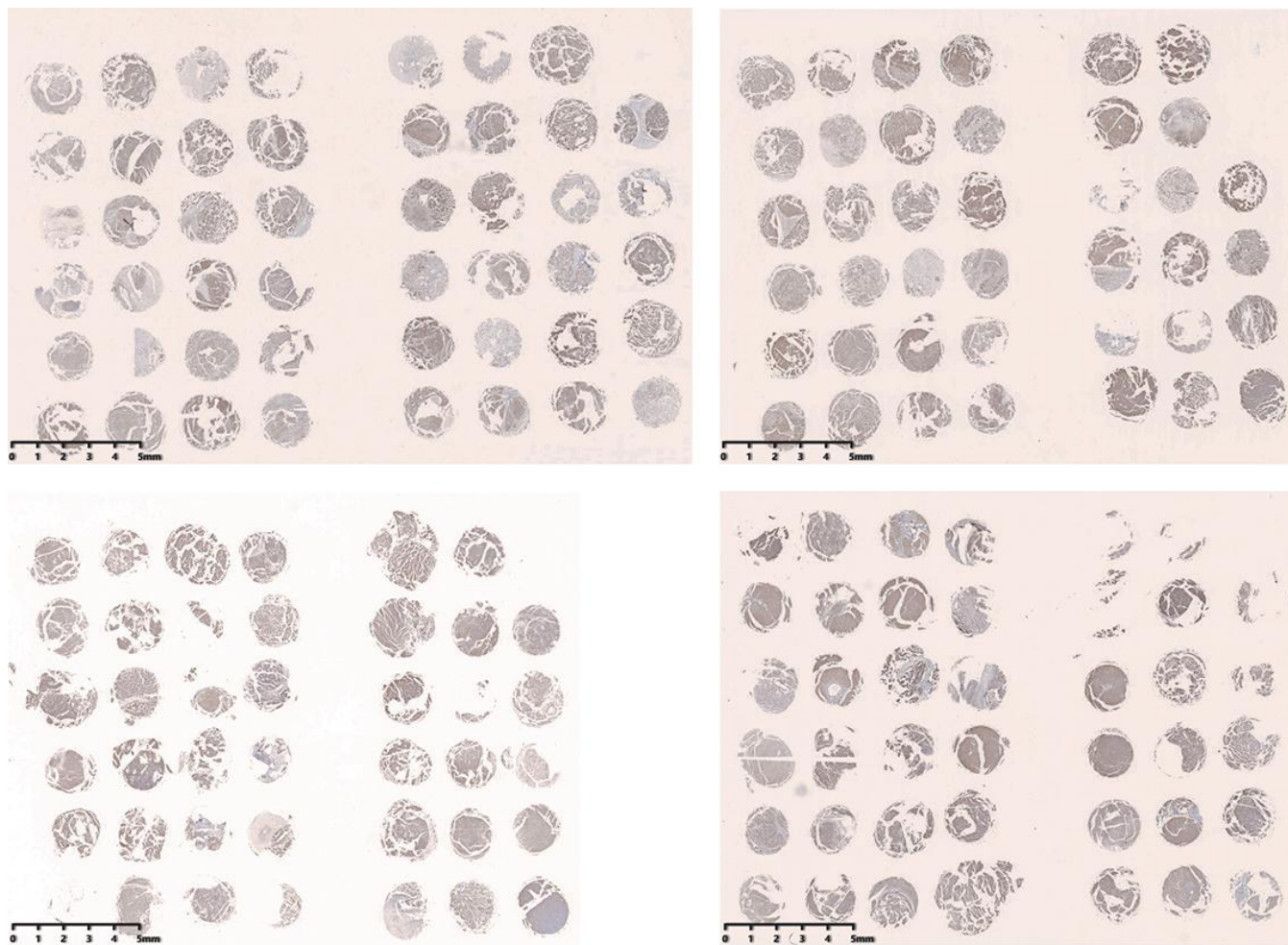

Supplementary Figure 1.

Supplement: Supplementary Figure 1 [file aging-11-102110-s002.pdf]
